# Standardized astragalus extract for attenuation of the immunosuppression induced by strenuous physical exercise: randomized controlled trial

**DOI:** 10.1186/s12970-021-00425-5

**Published:** 2021-07-16

**Authors:** Ewa Latour, Jaroslaw Arlet, Emilia E. Latour, Artur Juszkiewicz, Karolina Łuczkowska, Anita Marcinkiewicz, Piotr Basta, Jerzy Trzeciak, Bogusław Machaliński, Anna Skarpańska-Stejnborn

**Affiliations:** 1 University of Physical Education in Poznań, Branch in Gorzów Wlkp, Poznań, Poland; 2 Department of General Pathology, Pomeranian Medical University, Rybacka 1, 70-204 Szczecin, Poland

**Keywords:** Immunosuppression, Strenuous exercise, Supplementation, Astragalus Membranaceus Root

## Abstract

**Background:**

This paper aimed to verify how a supplementation of rower’s diet with Astragalus Membranaceus Root (AMR) modulated their immune system response to maximal physical exertion.

**Methods:**

The double-blind study included 18 members of the Polish Rowing Team assigned to the supplemented group (*n* = 10), and the placebo group (*n* = 8). The participants performed a 2000 m test on a rowing ergometer at the beginning and at the end of the six-week of intensive training camp during which the supplemented group received 500 mg of AMR. Blood samples were obtained prior to, 1 min after completing, and 24 h after the exertion test. The levels of interleukin 2 (IL2), interleukin 4 (IL4), interleukin 10 (IL10), interferon ɤ (IFN-ɣ), and lactic acid were determined. Subpopulations of T regulatory lymphocytes [CD4+/CD25+/CD127−] (Treg), cytotoxic lymphocytes [CD8+/TCRαβ+] (CTL), natural killer cells [CD3−/CD16+/CD56+] (NK), and TCRδγ-positive cells (Tδγ) were determined with flow cytometry.

**Results:**

After the camp, the initial NK and Treg levels sustained at the baseline, while Tδγ counts increased relative to the levels in the placebo group. In the supplemented subgroup, a decrease in IL2 level in reaction to maximal exertion clearly deepened while the change in IL-2/IL-10 level induced by the recovery after this exertion clearly increased, relative to the changes in the placebo group.

**Conclusions:**

AMR restored the immunological balance in strenuously trained athlets through a stabilization of NK and Treg cells with a positive trend in Tδγ towards Th1 response during restitution by cytokine IL2 modulation.

**Supplementary Information:**

The online version contains supplementary material available at 10.1186/s12970-021-00425-5.

## Introduction

It has been reported that while moderate physical exercise improves immunity [1], strenuous exercise may exert an opposite effect by activating immunological and endocrine mechanisms similar to those that occur during chronic stress, trauma, and sepsis [2, 3]. Maximal intensity of physical exertion typical of high-performance athletes [4] constitutes a stress factor that induces an “immunological response” in which both pro- and anti-inflammatory factors increase, remaining in dynamic balance. Weighing this equilibrium in favor of either side is unbeneficial for sustaining endocrine homeostasis; however, athletes subjected to extended and exhaustive exercise are especially prone to excessive shifting towards anti-inflammatory, so-called type-2 T helper cells (Th2) responses, which may be manifested by occurrences of Upper Respiratory Tract Infections (URTI) and eczema [5–11]. A dietary supplement with an immunomodulating capability may act as a gentle and safe incursion, restoring balance in the immune system of athletes [12].

Astragalus Membranaceus Root (AMR) extract is a non-toxic, bioactive substance with beneficial effects on the immune system [13, 14], visible, among other actions, in leveling out imbalances between cellular (Th1) and humoral (Th2) immunological responses [15–19]. Numerous studies on supplementation with herbal extracts exhibit the unique among other herbal supplements effect of AMR supplementation, that is a beneficial shift in the immunological balance towards Th1. Among others an increase in interferon gamma and a decrease in interleukin 4 (IL4) levels in asthmatic children [17] has been observed, as well as a decrease in Th2 response cytokines in mice with artificially induced asthma model [20], or, based on the increase in interleukin 2 (IL2) and decrease in IL4, in rats subjected to forced swimming and food restriction [21]. Athletes use AMR in their daily routine as an adaptogenic and anti-fatigue agent for improving endurance performance [22], however, its impact on their immune system has not yet been assessed.

To assess the Th1/Th2 immunological balance, we observed changes in counts of particular lymphoid cell subsets susceptible to the immuno-modulatory cytokines. The so-called cell-mediated immune response, which is crucial for protection against intracellular pathogens, is mediated by cytotoxic lymphocytes such as natural killer (NK) cells, cytotoxic T CD8+ cells (CTL), and TCRδγ-positive (Tδγ). T regulatory lymphocytes, called Tregs, are characterized by antigen-specific activation; they play an essential role in immune tolerance, as well as gaining control over an overly reactive immune system. They also synthesize IL10, an anti-inflammatory cytokine. On the other hand, when Tregs are inadequately activated, they may also mitigate the function and proliferation of effector cells involved in the specific and nonspecific immune responses, such as CTLs, NK cells, monocytes, macrophages, and dendritic cells [23–25], thus increasing the body’s susceptibility to infections [26–31].

T CD4+ lymphocytes include, alongside Treg, T helper lymphocytes, which can be further divided into Th1 and Th2 [32]. Th1 lymphocytes produce pro-inflammatory cytokines such as interferon gamma (IFNγ) or IL2, which are responsible for defense against intracellular pathogens. In preferable circumstances, the levels of Th1 response cytokines are counterbalanced by Th2 cytokines such as IL4 and IL10, which respectively promote IgE responses in atopy and act as an anti-inflammatory factor [33]. Imbalances in this system may be caused by external stimuli such as strenuous exercise.

Interactions between cytotoxic lymphocytes and Treg were investigated in numerous studies concerning various pathological conditions (e.g., autoimmune diseases and neoplasms) [26, 27, 34–38], while the problem of imbalances in immune cells in athletes appears in fewer publications. Expanding the general knowledge in this matter would be undeniably beneficial, as it has already been reported that repeated strenuous exercise may persistently alter absolute and relative sizes of lymphocyte subpopulations, causing a prevalence of Treg [6, 39, 40], which is at least in part responsible for the resulting immunosuppression [10], consequently decreasing the number of NK cells and CTLs [11, 41, 42]. It has been suggested [25, 35, 38, 43] that the evaluation of Treg to effector T cell (NK, Tδγ, CTL) ratio is a better indicator of suppressive effects of Treg on various effector cells than a simple determination of their counts; therefore, we also conducted an assessment of this index, describing it as Treg/Tδγ.

For the assessment of the change direction in Th1/Th2 balance, a determination of immuno-modulatory cytokines levels is also crucial, as they are interrelated. By increasing or decreasing Th1 response cytokines (e.g. INFγ, IL2), their effects can be attenuated by increase or amplified by decrease in the levels of Th2 response cytokines (e.g., IL4, IL10) [44, 45]. IL4 stimulates a shift in the immune system towards the Th2 response, increases antibody production, and inhibits differentiation into Th1 subsets. IL10 decreases IL2 and IFN-ɣ release [46, 47], simultaneously reinforces humoral and attenuates a cellular response of the body by inhibiting the proliferation of particular cytotoxic cells; the converse actions are attributed to the IL2 cytokine, which influences the Th1/Th2 balance, shifting it towards Th1 response. IFN-ɣ in turn stimulates the cellular response [48–50]. IL2/IL4 and IL2/IL10 ratios have been repeatedly used as markers of inflammation and tissue injury in Systemic Lupus Erythematosus (SLE), or as an indication of cytokine imbalance in vitiligo [51, 52], also in athletes [53].

In light of the beneficial actions of AMR on the immune system, we hypothesized that using it as a supplementation can maintain balance of the immunological response in endurance athletes. Immunological balance was assessed by determining Th1/Th2 balance, indicated by measuring levels of immuno-modulatory cytokines IL2, IL4, IL10, IFN-ɣ, and T regulatory lymphocytes [CD4+/CD25+/CD127–] (Treg), cytotoxic lymphocytes [CD8+/TCRαβ+] (CTL), natural killer cells [CD3−/CD16+/CD56+] (NK), and TCRδγ-positive (Tδγ), in a group of rowers subjected to diversified, moderate to high intensity training.

## Materials and methods

### Study population

The study included 18 men, all members of the Polish Rowing Team (14 heavy-weight and 4 light-weight rowers), during their preparation training camp before the World Rowing Championships. Basic characteristics and sport classes of the athletes, divided into the supplemented (*n* = 10) and the placebo (*n* = 8) subgroup, are presented in Table 1. The statistical uncertainty of differences in anthropometric parameters and training experience between supplemented and placebo subgroup did not exceed the value of 0.05.

**Table 1:** Basic characteristics of the study groups

Parameters	Supplemented subgroup		Placebo subgroup	
	Mean	SD	Mean	SD
Age (years)	21.4	0.91	20.4	1.02
Body mass (kg)	90.3	7.20	86.1	5.71
Body height (cm)	190.0	2.61	188.8	4.87
Duration of training (years)	7.6	1.50	6.5	1.91

It was also not found the differences in power output, total row time over a 2000 m distance, or pre- and post-test LA levels (Table 2) between the groups with a statistical uncertainty lower than 0.05. The study was conducted between March and May during a long-term training camp, scheduled between the preparatory and competitive phases of the yearly training cycle. The characteristics of the training profile, such as intensity, volume (in minutes), and type (specific, i.e., rowing: endurance, technical, speed, etc., and nonspecific, i.e., jogging, strength) were recorded on a daily basis. The intensity of the training was classified based on the lactate acid (LA) threshold (4 mmol/L) as extensive (below the LA threshold), highly intensive (above the LA threshold), and extremely intensive (control tests) (Table 2).

**Table 2:** Training schedule for the week preceding blood sampling during the first and second examination

	Days before the first examination						
	1	2	3	4	5	6	7
Total training time, min/day	190	100	205	105	195	110	90
Time rowed, min/day	105	90	90	90	85	100	80
Distance rowed, km/day	20	18	18	20	18	20	16
Training for force development, min/day	–	–	90	–	70	–	–
Extensive endurance rowing training time, min/day	85	90	74	50	60	100	80
Highly intensive endurance rowing training time, min/day	20	–	16	40	25	–	–
Unspecific training (running, etc.), min/day	75	10	25	15	40	10	10
	Days before the second examination						
	1	2	3	4	5	6	7
Total training time, min/day	170	90	190	180	180	100	100
Time rowed, min/day	90	80	100	160	90	90	90
Distance rowed, km/day	20	16	20	36	20	18	18
Training for force development, min/day	–	–	80	–	70	–	–
Extensive endurance rowing training time, min/day	84	80	100	52	67	90	–
High intensity endurance rowing training time, min/day	64	80	100	115	76	90	90
Extremely intensive endurance rowing training time, min/day	26	–	–	45	24	–	–
Unspecific training (running, etc.), min/day	80	10	10	20	20	10	10

### Food intake

Throughout the study period, the athletes were accommodated at one of the Olympic Training Centers, where all of their meals were provided onsite. Their regular menu consisted of a mixed diet, providing the recommended dietary allowance (RDA) of carbohydrates, proteins, fats, and micronutrients (vitamins and minerals) in line with the Polish Nutrition Society guidelines [54]. Daily intakes of food, calories, fruits, and vegetables were the same throughout the entire study period.

The study subjects declared that they had ceased all drugs, medications, and dietary supplements at least 2 weeks prior to the study, and did not use them throughout the entire study period.

### Experimental procedure

Athletes who were randomly placed into the supplemented group (*n* = 10) received gelatin capsules with 500 mg astragalus root extract. The supplement is standardized to 0.5% 3-hydroxy 7-methoxy isoflavonoids. The excipients contained in the capsule were microcrystalline cellulose, as well as two anticaking agents: the calcium salts of fatty acids and silicon dioxide. The athletes randomly placed into the placebo group (*n* = 8) received visually identical capsules with cornstarch (500 mg per capsule).

The subjects were asked to take two capsules per day for a period of 6 weeks following the first exhaustion test.

### Training program

Training volumes (expressed in minutes per day) throughout the week preceding the first and second examinations, particularly for extensive rowing, intensive rowing, kilometers, and extensive non-specific training, are shown in Table 2. During the load-training phase (before the first examination), the total training volume amounted to 995 min/wk., including approximately 54.2% of extensive rowing, 18.6% of non-specific training (e.g., power training), and 10.2% of intensive rowing. Total training volume before the second examination was 1010 min/wk., and included approximately 60.9% of extensive rowing and 9.4% of intensive rowing.

### Rowing performance test

The athletes performed a controlled 2000 m time trial on the first day (prior to the supplementation) and at the end of training camp (after the supplementation). Each subject had to cover the 2000 m distance on a rowing ergometer (Concept II, USA) in as short time as possible (Table 3). Because the results of both tests were taken into consideration during the selection of the championship team, the athletes were highly motivated to perform both tests at maximal effort. Prior to each test, the subjects performed a 5-min individual warm-up on the ergometer.

**Table 3:** Changes in 2000 m rowing ergometer performance before and after supplementation

Parameters	Supplemented group (*n* = 10)				Placebo group (*n* = 8)			
	Before		After		Before		After	
	Mean	SD	Mean	SD	Mean	SD	Mean	SD
Power [Watt]	437	26.3	440	24.70	428	19.73	435	11.08
Power [W/kg]	4.84	0.22	4.89	0.30	4.82	0.29	4.94	0.18
LA_min_ [mmol/L]^a^	1.3	0.23	1.6	0.72	1.6	0.17	1.7	0.38
LA_max_ (mmol/L]^a^	15.8	4.04	14.7	2.83	17.2	2.66	15.5	3.25
Time [s]	371.6	8.05	370.4	7.19	374.8	5. 08	371.4	3.42

^a^LA – lactate acid. No statistically significant (*p* < 0.05) differences were found between the pre- and post-supplementation results

### Sample treatment

Blood samples from the antecubital vein were collected in test tubes with dipotassium ethylene diamine tetra-acetic acid (K2EDTA) as an anticoagulant. Blood was collected before each 2000 m test (after 7–8 h of overnight fast), 1 min after completing the test, and after a 24-h recovery period. Blood samples were taken for the analysis of white blood cells (WBC) as well as for the percentage of lymphocytes, monocytes, and granulocytes in a MYTHIC 18 Hematology Analyzer (Orphee Medical; Geneva, Switzerland). Blood was drawn from the vein of the elbow into 9 ml polyethylene tubes (to obtain serum) and centrifuged at 3000 rpm for 10 min. After removing the plasma and adding 1x Lysing Buffer (BD Biosciences), the samples were incubated in darkness for 15 min. A PBS buffer was then added, and the cells were washed twice to remove all erythrocytes. Blood samples were collected into test tubes without additives and after the centrifugation (20 min at 4000 rpm), the sera were stored in − 80 °C until the interleukin level analysis. Additionally, capillary blood samples from an ear lobe were collected prior to and after each exercise test to assess lactate acid (LA) levels.

### Measurements

Cytometric analysis of lymphocyte subpopulations: T regulatory lymphocytes [CD4+/CD25+/CD127–] (Treg), cytotoxic lymphocytes [CD8+/TCRαβ+] (CTL), natural killer cells [CD3−/CD16+/CD56+] (NK), and TCRδγ-positive (Tδγ) was conducted after their labeling with fluorochrome-conjugated antibodies from BD Biosciences. Cells obtained after a hypotonic lysis of peripheral blood were incubated in darkness at room temperature for 20 min with the respective antibody at a concentration specified by the manufacturer to identify each lymphocyte subpopulation (Table 3). Then, the cells were washed twice with PBS buffer and left in darkness in 3.7% formaldehyde solution for 10 min. Afterwards, the cells were again washed with a PBP buffer and 100 μL of DAPI solution (1 mg/mL, Thermo Fisher Scientific) was added to stain cell nuclei. The cells were incubated in darkness at room temperature for 5 min, washed twice, and suspended in 250 μL of PBS buffer. After labeling, the cells were analyzed with a LSRII flow cytometer from BD Biosciences, coupled with BD FACSDiva software. The results were expressed as the percentage of the analyzed cells in the total number of lymphocytes in the examined sample.

Serum IL2 (interleukin-2) was measured using a commercially available enzyme immunoassay (Human High Sensitivity, ELISA; Abcam, Cambridge, UK) with an assay range of 1.87–60 pg/ml. Serum concentrations of IL4 (in pg/ml) were quantified using a commercially available enzyme immunoassay (ELISA; Abcam, Cambridge, UK) with an assay range of 0.3–10 pg/ml. Serum IL10 was measured using a commercially available enzyme immunoassay (ELISA; Abcam, Cambridge, UK) with an assay range of 1.56–50 pg/ml. Serum concentrations of interferon gamma (IFN-γ) in pg/ml were quantified using a commercially available enzyme immunoassay (ELISA; Quantikine HS, R&D Systems; Minneapolis, USA) with an assay range of 15.6–1000 pg/ml.

The concentration of lactic acid (LA) in capillary blood was determined immediately after sampling using a commercially available kit (Dr Lange; Germany); lactic acid concentrations were expressed in mmol/l. Coefficients of variation for all assays were less than 13%.

### Statistical analysis

Statistical analyses and charts were performed and developed with the R Language and Environment for Statistical Computing [55]. The level of changes in the values of analyzed indices was estimated as the relative change:

1 $$ RC\left[\%\right]=\mathit{\ln}\left(\frac{x_2}{x_1}\right)\cdot 100 $$

where *x*_1_ = value before the change, and *x*_2_ = value after the change.

The statistical certainty of the observation of changes was estimated with the one-sample Cohen’s effect size index (*d*) and additionally assessed with the *p*-value of one-sample Welch’s T-test of the hypothesis about their mean being equal to zero.

**Fig. 1:**
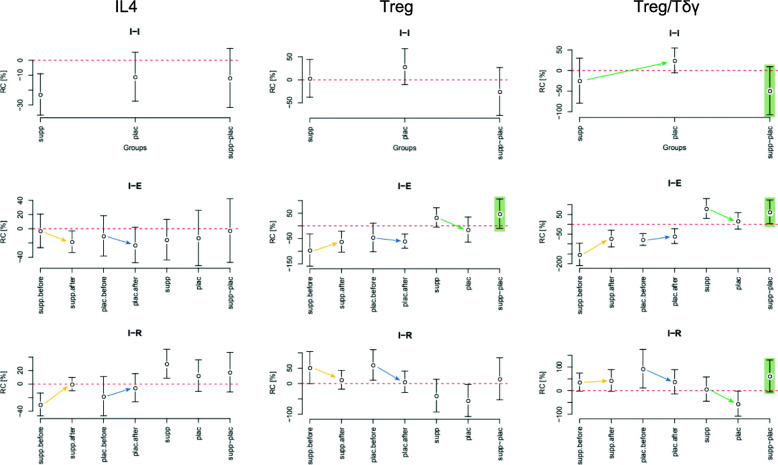
Relative changes in concentration of IL4, Treg, and Treg/Tδγ between initial states before and after training periods (I-I), between initial and after exertion states (I-E), and between initial and after restitution states (I-R). (I-I) levels of Treg were sustained with an intensive decrease of IL4 (I-I) in the supplemented subgroup, in contrast to an increase of Treg for the placebo subgroup. The post-camp Treg/Tδγ ratio (*d* = 0.86, *p* = 0.09) was relatively lower in the supplement subgroup as compared to placebo subgroup

**Fig. 2:**
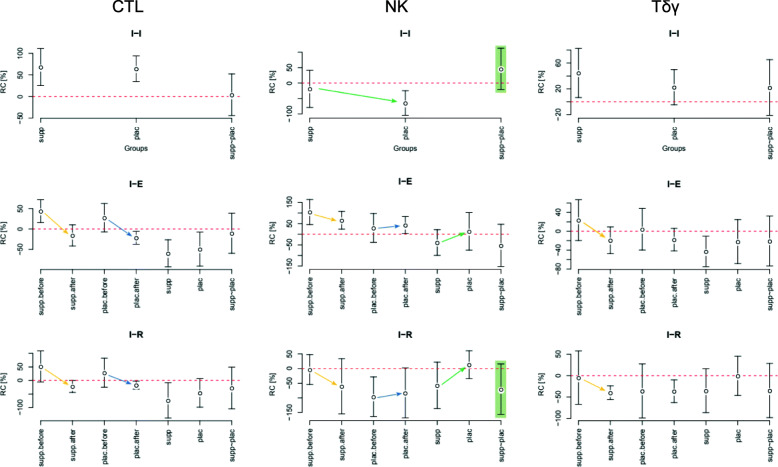
Relative changes in concentration of CTL, NK, and Tδγ between initial states before and after training period (I-I), between initial and after exertion states (I-E), and between initial and after restitution states (I-R). The before and after camp-training baseline levels of NK were sustained in the supplemented subgroup, in contrast to a decrease of NK for the placebo subgroup. The intensification trend in the supplemental Tδγ increased

**Fig. 3:**
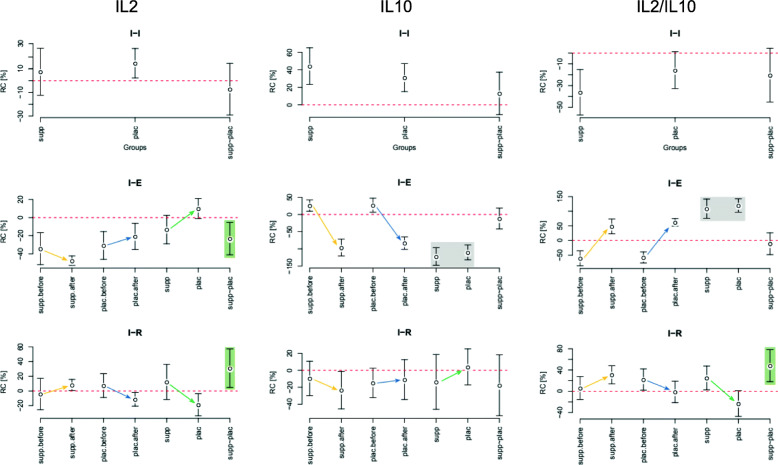
Relative changes in concentration of IL2, IL10, and IL2/IL10 between initial states before and after training period (I-I), between initial and after exertion states (I-E), and between initial and after restitution states (I-R). The directions of IL2 changes are mutually opposite in the subgroups and at both study points after exertion test (I-E – I-R). The changes in IL2 induced by *exertion* (I-E) are similar to the changes in IL10 induced by restitution (I-R); the mutual relations of these changes determine the changes in the IL2 / IL10 index. The post-exertion IL2 decrease (*d* = 1.22, *p* = 0.02) generates greater IL2/IL10 ratio (*d* = 1.61, *p* < 0.01) after recovery

**Fig. 4:**
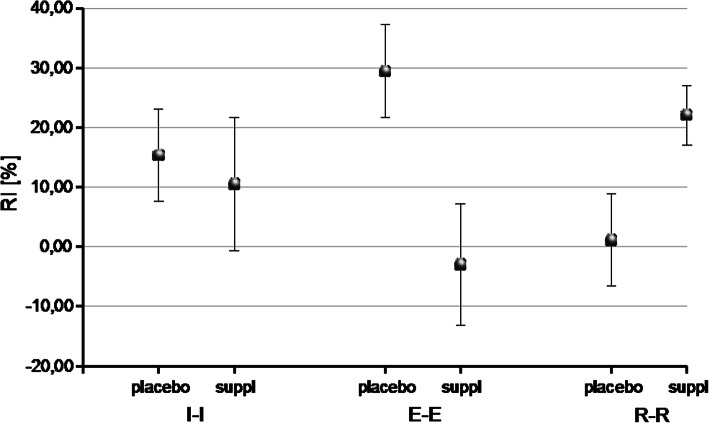
Relative changes in concentration of IL2 between initial (I-I), exertion (E-E), and restitution (R-R) states before and after training period, with their 95% confidence intervals. In the supplemented subgroup, absolute post-exertion IL2 levels (E-E) remained similar to those before the training, but increased in the control subgroup

The data sets were described with its mean along with 95% confidence intervals (CIs), the borders of which were presented in square brackets ([CI_lower_ CI_upper_]). The most important results are presented in text and in Figs. 1, 2, 3, 4. All results are presented in more detail in the supplementary materials, in tables S1–S20.

**Scheme A1:**
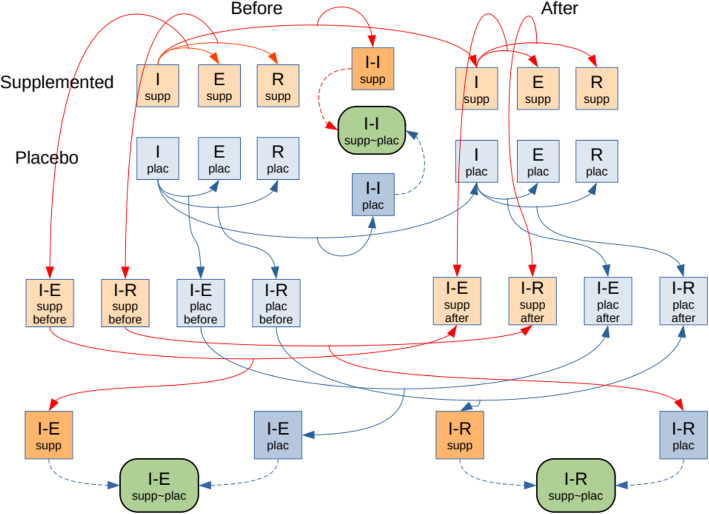
Diagram of analyses. The figures in the diagram represent: 1) the measurement data sets obtained in the initial state (I), after effort (E) and after restitution (R); 2) the relative changes between them (I-E, I-R) in one measuring session; 3) the relative changes between the 1st and the 2nd measuring session (I-I, I-E, I-R); and 4) the resulting differences between before-after relative changes or values in initial states for the supplemented and the placebo group (green ovals)

Scheme A1 in Appendix presents a diagram of analyses with the names of the variables quoted in Figs. 1, 2, 3.

## Results

A non-trivial difference between experimental and control subgroups was observed in NK [CD3−/CD16+/CD56+] and Treg [CD4+/CD25+/CD127−] lymphocytes, as well as the Treg/Tδγ ratio, IL2 and IL2/IL10 ratio in the context of several post-exertion changes among the factors considered. Primarily, in the supplemented group, a stabilizing effect of initial levels of NK, Treg and IL2 was visible, along with different responses to the severe exertion (I-E) in NK and IL2 in both subgroups. Changes were also noted in IL2, IL10, IL2/IL10 ratio, NK, Treg, and Treg/Tδγ ratios as a result of the recovery (I-R).

All analyzed parameters exhibited noticeable changes imposed with both acute exertion and long-term training. After the training camp, initial levels of IL2, IL10, CTL, and Tδγ increased while IL2/IL10 ratio and Treg counts decreased. Acute exertion imposed changes in all of the analyzed parameters, both directly after the exertion test (I-E) and after recovery (I-R). In cytokines (I-E), changes were followed by the return to the baseline during recovery (I-R); in lymphocytes, the post-exertion alteration was also sustained through recovery. The direction of changes in IL10, CTL, and Tδγ imposed by exertion was reversed after the camp due to the elevation of initial values of these parameters.

### Cytometry

The supplemented subgroup sustained the before and after camp-training baseline levels of NK and Treg, in contrast to a decrease of NK and an increase of Treg in the placebo subgroup (Figs. 1 and 2). Due to the parallel increase in the initial Treg levels that occurred only in the control subgroup, with the simultaneous intensification of the supplemental Tδγ increase, the post-camp Treg/Tδγ ratio was relatively lower in the supplement subgroup as compared to the placebo subgroup (*d* = 0.86, *p* = 0.09).

Among the tested lymphocyte subpopulations, the CTL were the most numerous and their count increased the most after the camp, which was not influenced by the supplementation (average initial CTL level increased to a similar degree above 60% in both subgroups). The effect of the supplementation was the most visible in the second largest of the observed lymphocyte subpopulations, NK, which notably (*d* = 1.16, *p* = 0.01) decreased after the camp in the placebo subgroup (average initial NK value decreased by −64 [−104.42 –24.71]%), which did not occur in the supplemented subgroup. A similar observation on the influence of the supplement was made on the Tδγ lymphocytes, which increased in both subgroups, but more prominently in the supplemented subgroup (*d* = 0.98, *p* = 0.03).

### Cytokines

Both acute exertion and the long-term training camp imposed noticeable changes in IL2, IL4, and IL10, but not in IFN-ɣ. Changes in the initial level are more or less visible in both subgroups but exhibit a similar change direction: an increase in IL2, along with a decrease in IL4 and IL2/IL10 ratio. The effect of the supplementation was most evident in the differentiation of IL2 levels between subgroups (Fig. 3). Before the camp and in response to acute exertion (I-E), a strong decline in IL2 (−34 [−52 –17]% in supplemented and −31 [−46 –16]% in control subgroup) was occurring concomitantly with an increase in IL10 (25.87 [9.49 42.25]% in supplemented and 26.75 [6.25 47.24]% in control subgroup). As a result of the training camp, initial IL2 levels (I) increased markedly only in the control subgroup (15 [2 27]%), while initial IL10 levels increased in all participants, regardless of supplementation (44.21 [23.32 65.10]% in supplemented and 31.10 [15.10 47.11]% in control subgroup); therefore, the IL2/IL10 ratio decreased more in the supplemented group relative to the control subgroup (*d* = 0.83, *p* = 0.1). In relation to the previously mentioned the prominent rise of initial IL10 levels in the second examination, the post-exertion change direction for this cytokine reversed: a decrease instead of an increase was noted, which manifested in a reversion of the direction of change in the IL2/IL10 ratio.

Through comparison of both subgroups of subjects, the reversed IL2 change direction was observed both directly after exertion (I-E) (*d =* 1.33, *p* = 0.01) and post-recovery (I-R) (*d =* 1.22, *p* = 0.02): comparing to the results from the first examination, the decrease in reaction to the exertion was greater in the experimental subgroup and smaller in the placebo subgroup. The situation was diverted for the recovery (Fig. 3). The aforementioned differentiation after long-term camp training occurred because the absolute post-exertion IL2 levels (E-E) remained similar as before the training in the supplemented subgroup, but increased by approximately 26% in the control subgroup (Table 4, Fig. 4). This difference, combined with the post-training increase of the initial IL2 levels observed in all participants, resulted in a difference in the I-E decrease between both subgroups, for which the I-E decrease in the supplemented subgroup was greater and in the placebo subgroup it was smaller. The similar between-session differences were also visible for IR of IL10 and in consequence for IR of IL2/IL10 ratio, which was outstandingly raised during recovery after the second test in the supplemented group, and decreased in the control group, resulting in between-group relative change of 48.28 [18.05 78.51]%, (d = 1.61, *p* < 0.005).

**Table 4:** Relative changes in concentration of IL-2 in the initial (I-I), post-exertion (E-E), and restitution (R-R) states between before- and after training period

	I-I			E-E			R-R		
Group	RC [%]	d	p	RC[%]	d	p	RC[%]	d	p
Placebo	15,33 [7,57 23,08]	−0,21	0,68	29,43 [21,68 37,19]	−1,51	0,01	1,11 [−6,64 8,86]	1,37	0,02
Supplemented	10,49 [−0,74 21,73]			−3,00 [−13,24 7,24]			22,07 [17,07 27, 08]		

The levels of analyzed cytokines and leukocytes did not correlate to each other to a noticeable degree.

## Discussion

To evaluate the immunomodulatory effect of AMR supplementation, cytokine and cytometry parameters of immunological balance were analyzed in a group of rowers from the National Polish Team who were adapted to a very intensive training and exceptionally high lactic acid levels as a result (Table 3). It was presumed that the supplement intake would maintain the immunological balance, disordered by fatigue involved in both bouts of acute exertion and the long duration of intensive camp training. The research was conducted with seemingly small groups of participants that constitute actually a large part of the population of elite rowers, selected in terms of physical performance and training experience, similar age and body build, the same operating environment, a similar lifestyle, and diet. This strict selection minimized the influence of these factors on the analysis result. The consistency of the results achieved thanks to this made it possible to consider them as reliable. Perhaps, if the physiological models of changes in components extracted from the tissues in response to exertion were known, it could be possible to obtain more accurate results, with the use of based on these models indices of change. However, the more general indicator of relative change used in the research (RC) allowed a reliable analysis.

The immuno-stabilizing effect of AMR was visible in the stabilization of base (initial) levels of numerous parameters, along with the shift of the immunological balance towards Th1 response after the second exertion test, crowning the six-week supplementation period. Our main observation was that in the supplemented subgroup, long-term training did not cause visible changes in the NK [CD3/CD16+/CD56+] initial percentage, in contrast to the placebo subgroup, for whom the percentage clearly decreased (RC = −65 [−104 –25]%). Furthermore, Tδγ levels increased (RC = 45 [7 83]%), while the Treg percentage did not change significantly, which consequently decreased the value of the Treg/Tδγ ratio, relative to the control group (RC = 51.98 [−113.07 9.12]%, *d =* 0.86). Analogically, relative to the control group, the long-term training camp also resulted in a post-recovery (I-R) increase in IL2/IL10 ratio (RC = 48.28 [18.05 78.51], *d =* 1.61) as a consequence of post-exertion (I-E) IL2 decrease (RC = −23.2 [−41.02 –5.37], *d =* 1.33).

After the camp, an increase in the initial levels of IL2, IL10, CTL, and Tδγ with a decrease in IL2/IL10 ratio and IL4 were also observed, thus suggests a general activation of the immune system [4]. During the second examination, the initial (I) CTL [CD8+/TCRαβ+] percentage increased to a similar degree both in the supplemented (RC = 68 [25 111]%) and the control (RC = 64 [34 94]%) subgroup – the greatest observed among all analyzed cell populations. This reveals the impact of the long term intensive training itself on enhancing the number of CTL cells, which was not modifiable by AMR supplementation. On the other hand, NK notably decreased in the placebo subgroup, while in the supplemented subgroup, their average level remained at the baseline. This could be considered a protective effect of the AMR supplementation on NK levels, preventing their decrease under the impact of Treg and strenuous exercise. Similarly, Tδγ lymphocytes increased in both subgroups (RC = 44.70 [6.70 82.71]% supplemented and 22.64 [−4.58 49.87]% placebo).

CTL, NK, and Tδγ cells are the WBC subsets representative of the Th1, cell-mediated immune response. They are responsible for cytotoxicity, which is known to be a powerful stressor able to shift the Th1/Th2 balance towards the Th2, but it may have been impaired due to strenuous exercise [6]. As observed in our study, the effects of AMR supplementation on NK and Tδγ cells were influential, helping sustain the balance between the cytotoxic lymphocytes and the Treg. The I-I difference in initial Treg lymphocyte counts as well as Treg/Tδγ ratios between the first and second examinations increased more significantly in the control than in the supplemented subgroup. This observation means that AMR may prevent the cellular immunological response from weighting in favor of the Th2 response in circumstances of strenuous exercise stimulation.

Despite the beneficial Th1 shift observed in cytometry of supplemented athletes, their IL2/IL10 ratio presented relatively lower values, characteristic of Th2 response. The lowering of the post-camp initial (I-I) IL2/IL10 ratio in both subgroups indicates a shift towards Th2 response (Fig. 1); this index was lower in the supplemented subgroup than in control, which is a consequence of the lack of a clear increase of initial IL2, accompanied by increase of IL10. During the recovery period after exertion, however, when athletes are the most susceptible to URTI due to immunosuppression [56], the supplemented athletes attained higher values of both IL2 and IL2/IL10 ratio with respect to control group, which further demonstrates the beneficial immune stabilizing actions of AMR. The after recovery increase in IL2/IL10 ratio in the supplemented subgroup observed after the second examination occurred by an experimental differentiation of IL2’s reaction to exertion which in turn shaped a converse differentiation of the changes, thus restoring the balance during rest (I-R). This reaction was strong enough to be mirrored in the similar I-R changes in IL2/IL10 ratio.

During the second examination, after the camp, the post-exertion I-E decrease in IL2 was greater in the supplemented group in respect to the controls; in the control subgroup, the decrease was less significant as compared to the first examination as a result of elevated post-camp initial IL2 values. In the supplemented subgroup, this enabled a reproduction of post-exertion IL2 levels similar to the values from the first examination, followed by an adequately fast return to the baseline during rest, resulting in a larger I-R increase that suggests greater dynamics of changes in IL2 levels. Even though I-E values of IL2 after the camp decreased more in the supplemented subgroup and less in the control, it did not modify the I-E, but rather the I-R values for IL2/IL10 ratio according to the reports about the connection between the two. Post-recovery IL10 change trends seem to reproduce the post-exertion IL2 change trends; while IL2 in the supplemented subgroup decreased after exertion, IL10 decreased during recovery, which, along with the I-R IL2 increase, resulted in elevation of IL2/IL10 ratio, thus indicating a shift towards Th1 response.

The post-exertion decrease observed in IL2 levels, followed by its increase back to the baseline, was noted in both examinations; in the second examination, however, said decrease was greater in the supplemented subgroup, as compared to both the control subgroup and the results of the first examination. Such a set of changes suggests a transient shift in immunological balance towards Th2 response resulting from exertion, followed by restoration of Th1 response intensity, which was reduced in placebo subgroup and sustained in the supplemented subgroup after long-term training.

A previous study [57] revealed a crucial role of IL2 in the efficient suppression of T effector cells by Tregs. Since Tregs themselves do not synthesize IL2, their function depends on levels of this cytokine paracrinally released by other particular cells. The results suggest that Treg compete for IL2 with T effector cells, suppressing their function by consumption of this crucial cytokine. Therefore, even though high doses of IL2 make T effector cells immune to suppression by Treg, certain levels of this cytokine are crucial for Treg immunosuppressive function and priming their IL10 synthesis. This hypothesis seems to be in line with data obtained from our study, and it would explain why, as compared to the supplemented subgroup, the control was characterized by a greater post-camp I-I increase in IL2, a cytokine mainly described as typical of Th1 response, while the immunological balance of the counts of assessed cells seemed to be shifted towards Th2 response. It could also explain the relation between IL2 and IL10 cytokines: if IL2 primes IL10 synthesis by Treg, then lower post-exertion IL2 levels in the supplemented subgroup could contribute to a post-recovery decrease in IL10.

Another manifestation of sustained immunological balance was also immunosuppression prevention, as observed through a lower Treg/Tδγ ratio in the supplemented subgroup. Therefore, the post-exertion shift towards Th2 response had a different character in both subgroups: in the control subgroup it was clearly visible, as seen in level changes of immunologically active cells, while it was mostly observed in cytokine changes in the supplemented subgroup. This indicates that AMR has adaptogenic effects, expressed as a stabilization of immunological parameters, along with the maintenance of essential levels of Th1-related cytotoxicity in reaction to exertion despite the general tendency towards a Th2 response.

Our study on AMR intake in athletes demonstrates a beneficial shift of Th1/Th2 balance that is in line with existing articles on the use of this supplement. Although it was observed on a basis of different indices, compositional observations are partially convergent. Captured among our observations was a larger resting increase in IL2/IL10 ratio along with an increase in IL2, a cytokine typical of Th1 response; this is consistent with an earlier study, which reported an increase in IL2 in AMR supplemented rats subjected to forced swimming and food restriction [21], but it opposes a study on asthma model in mice, in which Th1/Th2 change resulted from a decrease in Th2 response cytokines [20].

In this study, the supplement did not modify the IL4 or IFN-ɣ reaction, which has been reported by other authors [17, 21]. The diversity of immune response models and stressors [58] could explain this; in our case, for example, we noted that a clear lack of changes in IFN-ɣ is typical of strenuous exercise [53]. We also observed that the cytokine profile depends presumably on the load type, as numerous other works indicate [59]. Changes in other blood parameters have also been presented in articles concerning exertion [60]. Reduced blood lactic acid levels were noted in the supplemented athletes, which is in line with animal studies on AMR intake [22, 61].

The discovered phenomenon of maintaining the immune balance in a group of heavily-loaded athletes provides science-based evidence on the toning effect on the immune system, exceptional for Astragalus among other herbal supplements. Although further research is required to confirm this advantage, it seems that the protection against illnesses typical for shifting immunological balance towards Th2, such as URTI or eczema, could therefore be attained by the implementation of the AMR extract into athletes’ diet. This discovery also justifies undertaking the work on the dosage and expanding the knowledge regarding the supplement’s active mechanisms by adding certain cytokines not involved in this introductory paper to the panel of diagnostic tests (e.g. TNF, IL17, IL1, IL6).

## Conclusions

AMR stabilizes the immune system in athletes by restoring the immunological balance towards Th1 response during restitution after strenuous exercise. Adaptogenic effect consisted in stabilisation of NK [CD3−/CD16+/CD56+] and Treg [CD4+/CD25+/CD127−] levels with the positive trend of Tδγ [TCRδγ-positive] shaping the decrease in Treg/Tδγ ratio, along with maintenance of post-exertion IL2 decrease generating greater IL2/IL10 ratio after recovery. The discovered immuno-tonic effect gives the hope to use AMR in athletes’ daily routine to protect them against Th2-shift related illnesses.

### Supplementary Information

**Additional file 1.**

## Data Availability

The datasets during and/or analysed during the current study available from the corresponding author on reasonable request.

## Abbreviations

AMR: Astragalus Membranaceus Root
IFN-ɣ: Interferon ɣ
I-E: Relative change from initial to after-exertion state
LA: Lactate acid
RDA: Recommended dietary allowance
TAC: Total antioxidant capacity
URTI: Upper Respiratory Tract Infections
CTL: Cytotoxic lymphocytes
IL2: 4, 10, Interleukin 2, 4, 10
I-R: Relative change from initial to after- recovery state
NK: Natural killer cells
Tδγ: TCRδγ-positiv cells, T-cell receptor δγ positiv cells
Treg: Regulatory lymphocytes
WBC: White blood cells
