# Supplementary material for: Standardized astragalus extract for attenuation of the immunosuppression induced by strenuous physical exercise: randomized controlled trial
Source: J Int Soc Sports Nutr. 2021 Jul 16;18:57. doi: 10.1186/s12970-021-00425-5 (PMC8285774; doi:10.1186/s12970-021-00425-5)
Supplement: Supplementary file 1 — Additional file 1. [file 12970_2021_425_MOESM1_ESM.doc]

Table S1. Concentration of IFN‑γ [pg/ml] before and after the camp training and its percentage change in initial states caused by this training.

|  |  | before |  | after |  | after − before | | |
| --- | --- | --- | --- | --- | --- | --- | --- | --- |
| set | state | mean |  | mean |  | RC [%] | d | p |
| supp | I | 1.00 [0.85 1.14] |  | 0.94 [0.83 1.06] |  | -5.09 [-14.44 4.26] | -0.46 [-1.29 0.38] | 0.24 |
|  | E | 1.01 [0.88 1.14] |  | 0.98 [0.59 1.36] |  |  |  |  |
|  | R | 0.90 [0.79 1.02] |  | 0.85 [0.80 0.91] |  |  |  |  |
| plac | I | 1.01 [0.92 1.10] |  | 0.97 [0.86 1.07] |  | -4.09 [-19.45 11.26] | -0.19 [-0.91 0.52] | 0.56 |
|  | E | 1.00 [0.91 1.09] |  | 0.74 [0.60 0.89] |  |  |  |  |
|  | R | 0.94 [0.83 1.04] |  | 0.90 [0.78 1.03] |  |  |  |  |
| supp − plac | I |  |  |  |  | -1 [15.84 -0.06] | -0.06 [-1.08 0.96] | 0.9 |

Table S2. Relative changes of IFN‑γ concentration induced by excursion and by restitution and the differences in these changes between after‑ and before‑training measurement sessions along with Cohen’s d effect size and the probability of hypothesis about equality of their means.

|  |  | before | | |  | after | | |  | after − before | | |
| --- | --- | --- | --- | --- | --- | --- | --- | --- | --- | --- | --- | --- |
| set | change | RC [%] | d | p |  | RC [%] | d | p |  | RC [%] | d | p |
| supp | I-E | 1.45 [-19.23 22.13] | 0.06 [-0.78 0.89] | 0.87 |  | -18.71 [-105.90 68.48] | -0.18 [-1.02 0.66] | 0.63 |  | -20.16 [-95.18 54.87] | -0.22 [-1.06 0.61] | 0.55 |
|  | I-R | -9.18 [-30.86 12.50] | -0.35 [-1.19 0.48] | 0.35 |  | -9.46 [-24.80 5.87] | -0.52 [-1.35 0.32] | 0.19 |  | -0.28 [-18.88 18.31] | -0.01 [-0.85 0.82] | 0.97 |
| plac | I-E | -0.81 [-9.36 7.74] | -0.07 [-0.78 0.65] | 0.84 |  | -29.40 [-55.74 -3.06] | -0.80 [-1.51 -0.08] | 0.03 |  | -28.59 [-58.29 1.11] | -0.69 [-1.40 0.03] | 0.06 |
|  | I-R | -7.76 [-22.71 7.20] | -0.37 [-1.09 0.34] | 0.27 |  | -7.58 [-29.46 14.31] | -0.25 [-0.96 0.47] | 0.45 |  | 0.18 [-33.62 33.98] | 0.00 [-0.71 0.72] | 0.99 |
| supp − plac | I-E |  |  |  |  |  |  |  |  | 8.43 [-68.75 85.62] | 0.12 [-0.95 1.18] | 0.81 |
|  | I-R |  |  |  |  |  |  |  |  | -0.46 [-36.84 35.92] | -0.01 [-1.03 1.01] | 0.98 |

Table S3. Concentration of IL2 [pg/ml] before and after the camp training and its percentage change in initial states caused by this training.

|  |  | before |  | after |  | after − before | | |
| --- | --- | --- | --- | --- | --- | --- | --- | --- |
| set | state | mean |  | mean |  | RC [%] | d | p |
| supp | I | 0.99 [0.83 1.15] |  | 1.06 [0.95 1.16] |  | 7.49 [-12.40 27.38] | 0.31 [-0.52 1.15] | 0.40 |
|  | E | 0.71 [0.59 0.83] |  | 0.66 [0.56 0.76] |  |  |  |  |
|  | R | 0.94 [0.86 1.02] |  | 1.14 [1.10 1.17] |  |  |  |  |
| plac | I | 0.98 [0.87 1.10] |  | 1.13 [1.07 1.20] |  | 14.71 [2.23 27.18] | 0.84 [0.13 1.56] | 0.03 |
|  | E | 0.74 [0.60 0.88] |  | 0.93 [0.81 1.05] |  |  |  |  |
|  | R | 1.06 [0.92 1.21] |  | 1.02 [0.91 1.13] |  |  |  |  |
| supp − plac | I |  |  |  |  | -7.22 [14.6 -0.34] | -0.34 [-1.37 0.69] | 0.49 |

Table S4. Relative changes of IL2 concentration induced by excursion and by restitution and the differences in these changes between after‑ and before‑training measurement sessions along with Cohen’s d effect size and the probability of hypothesis about equality of their means.

|  |  | before | | |  | after | | |  | after − before | | |
| --- | --- | --- | --- | --- | --- | --- | --- | --- | --- | --- | --- | --- |
| set | change | RC [%] | d | p |  | RC [%] | d | p |  | RC [%] | d | p |
| supp | I-E | -34.31 [-51.95 -16.68] | -1.63 [-2.46 -0.79] | 0.00 |  | -47.55 [-52.78 -42.31] | -7.59 [-8.43 -6.76] | 0.00 |  | -13.23 [-28.98 2.51] | -0.70 [-1.54 0.13] | 0.09 |
|  | I-R | -4.18 [-25.57 17.20] | -0.16 [-1.00 0.67] | 0.66 |  | 8.07 [0.46 15.68] | 0.89 [0.05 1.72] | 0.04 |  | 12.25 [-11.63 36.13] | 0.43 [-0.41 1.26] | 0.26 |
| plac | I-E | -30.79 [-46.04 -15.53] | -1.44 [-2.16 -0.73] | 0.00 |  | -20.82 [-35.25 -6.39] | -1.03 [-1.75 -0.32] | 0.01 |  | 9.96 [-1.20 21.13] | 0.64 [-0.08 1.35] | 0.07 |
|  | I-R | 7.30 [-8.95 23.55] | 0.32 [-0.39 1.04] | 0.34 |  | -11.43 [-20.81 -2.05] | -0.87 [-1.59 -0.16] | 0.02 |  | -18.73 [-33.72 -3.74] | -0.89 [-1.61 -0.18] | 0.02 |
| supp − plac | I-E |  |  |  |  |  |  |  |  | -23.2 [-41.02 -5.37] | -1.33 [-2.35 -0.31] | 0.01 |
|  | I-R |  |  |  |  |  |  |  |  | 30.98 [4.78 57.18] | 1.22  [0.19 2.25 ] | 0.02 |

Table S5. Concentration of IL4 [pg/ml] before and after the camp training and its percentage change in initial states caused by this training.

|  |  | before |  | after |  | after − before | | |
| --- | --- | --- | --- | --- | --- | --- | --- | --- |
| set | state | mean |  | mean |  | RC [%] | d | p |
| supp | I | 1.28 [1.15 1.40] |  | 1.02 [0.89 1.15] |  | -22.89 [-36.78 -9.00] | -1.38 [-2.21 -0.54] | 0.01 |
|  | E | 1.27 [1.01 1.53] |  | 0.86 [0.72 1.00] |  |  |  |  |
|  | R | 0.95 [0.81 1.10] |  | 1.03 [0.87 1.18] |  |  |  |  |
| plac | I | 1.22 [1.05 1.39] |  | 1.10 [0.94 1.25] |  | -11.03 [-27.39 5.33] | -0.48 [-1.20 0.23] | 0.16 |
|  | E | 1.17 [0.84 1.49] |  | 0.88 [0.73 1.03] |  |  |  |  |
|  | R | 1.04 [0.84 1.23] |  | 1.03 [0.93 1.13] |  |  |  |  |
| supp − plac | I |  |  |  |  | -11.86 [7.9 -0.6] | -0.6 [-1.61 0.4] | 0.22 |

Table S6. Relative changes of IL4 concentration induced by excursion and by restitution and the differences in these changes between after‑ and before‑training measurement sessions along with Cohen’s d effect size and the probability of hypothesis about equality of their means.

|  |  | before | | |  | after | | |  | after − before | | |
| --- | --- | --- | --- | --- | --- | --- | --- | --- | --- | --- | --- | --- |
| set | change | RC [%] | d | p |  | RC [%] | d | p |  | RC [%] | d | p |
| supp | I-E | -2.97 [-26.64 20.70] | -0.10 [-0.94 0.73] | 0.78 |  | -18.37 [-33.62 -3.12] | -1.01 [-1.84 -0.17] | 0.02 |  | -15.40 [-43.97 13.16] | -0.45 [-1.29 0.39] | 0.24 |
|  | I-R | -30.17 [-47.01 -13.33] | -1.50 [-2.33 -0.66] | 0.00 |  | -0.02 [-9.91 9.87] | 0.00 [-0.84 0.83] | 1.00 |  | 30.15 [8.72 51.57] | 1.18 [0.34 2.01] | 0.01 |
| plac | I-E | -10.09 [-38.38 18.21] | -0.25 [-0.97 0.46] | 0.44 |  | -22.98 [-47.84 1.88] | -0.66 [-1.38 0.05] | 0.07 |  | -12.89 [-51.91 26.13] | -0.24 [-0.95 0.48] | 0.47 |
|  | I-R | -17.94 [-46.92 11.03] | -0.44 [-1.16 0.27] | 0.19 |  | -5.38 [-26.15 15.40] | -0.19 [-0.90 0.53] | 0.57 |  | 12.57 [-10.75 35.88] | 0.39 [-0.33 1.10] | 0.25 |
| supp − plac | I-E |  |  |  |  |  |  |  |  | -2.51 [-47.32 42.3] | -0.06 [-1.07 0.95] | 0.91 |
|  | I-R |  |  |  |  |  |  |  |  | 17.58 [-11.51 46.67] | 0.61  [-0.4 1.61] | 0.22 |

Table S7. Concentration of IL10 [pg/ml] before and after the camp training and its percentage change in initial states caused by this training.

|  |  | before |  | after |  | after − before | | |
| --- | --- | --- | --- | --- | --- | --- | --- | --- |
| set | state | mean |  | mean |  | RC [%] | d | p |
| supp | I | 1.41 [1.18 1.64] |  | 2.21 [1.81 2.60] |  | 44.21 [23.32 65.10] | 1.77 [0.93 2.61] | 0.00 |
|  | E | 1.80 [1.67 1.94] |  | 0.84 [0.72 0.96] |  |  |  |  |
|  | R | 1.27 [1.15 1.39] |  | 1.75 [1.42 2.07] |  |  |  |  |
| plac | I | 1.42 [1.22 1.63] |  | 1.92 [1.75 2.09] |  | 31.10 [15.10 47.11] | 1.39 [0.67 2.11] | 0.00 |
|  | E | 1.83 [1.69 1.97] |  | 0.84 [0.72 0.97] |  |  |  |  |
|  | R | 1.21 [1.12 1.30] |  | 1.76 [1.45 2.07] |  |  |  |  |
| supp − plac | I |  |  |  |  | 13.11 [37.34 0.55] | 0.55 [-0.47 1.56] | 0.27 |

Table S8. Relative changes of IL10 concentration induced by excursion and by restitution and the differences in these changes between after‑ and before‑training measurement sessions along with Cohen’s d effect size and the probability of hypothesis about equality of their means.

|  |  | before | | |  | after | | |  | after − before | | |
| --- | --- | --- | --- | --- | --- | --- | --- | --- | --- | --- | --- | --- |
| set | change | RC [%] | d | p |  | RC [%] | d | p |  | RC [%] | d | p |
| supp | I-E | 25.87 [9.49 42.25] | 1.32 [0.48 2.16] | 0.01 |  | -96.21 [-120.59 -71.82] | -3.30 [-4.13 -2.46] | 0.00 |  | -122.08 [-147.48 -96.68] | -4.02 [-4.85 -3.18] | 0.00 |
|  | I-R | -9.61 [-29.91 10.70] | -0.40 [-1.23 0.44] | 0.30 |  | -23.35 [-45.42 -1.28] | -0.88 [-1.72 -0.05] | 0.04 |  | -13.74 [-46.08 18.60] | -0.36 [-1.19 0.48] | 0.35 |
| plac | I-E | 26.75 [6.25 47.24] | 0.93 [0.22 1.65] | 0.02 |  | -83.33 [-101.29 -65.38] | -3.32 [-4.04 -2.60] | 0.00 |  | -110.08 [-131.44 -88.72] | -3.69 [-4.40 -2.97] | 0.00 |
|  | I-R | -14.88 [-32.28 2.52] | -0.61 [-1.33 0.10] | 0.09 |  | -10.96 [-34.46 12.54] | -0.33 [-1.05 0.38] | 0.32 |  | 3.92 [-17.35 25.19] | 0.13 [-0.58 0.85] | 0.69 |
| supp − plac | I-E |  |  |  |  |  |  |  |  | -12 [-42.48 18.48] | -0.4 [-1.41 0.61] | 0.41 |
|  | I-R |  |  |  |  |  |  |  |  | -17.66 [-53.53 18.22] | -0.5 [-1.53 0.52] | 0.31 |

Table S9. The ratio of IL2 / IL10 before and after the camp training and its percentage change in initial states caused by this training.

|  |  | before |  | after |  | after − before | | |
| --- | --- | --- | --- | --- | --- | --- | --- | --- |
| set | state | mean |  | mean |  | RC [%] | d | p |
| supp | I | 0.71 [0.61 0.81] |  | 0.50 [0.42 0.57] |  | -36.17 [-57.20 -15.13] | -1.44 [-2.27 -0.60] | 0.00 |
|  | E | 0.39 [0.32 0.46] |  | 0.81 [0.65 0.97] |  |  |  |  |
|  | R | 0.75 [0.67 0.83] |  | 0.68 [0.55 0.81] |  |  |  |  |
| plac | I | 0.70 [0.64 0.77] |  | 0.61 [0.52 0.69] |  | -15.82 [-32.85 1.20] | -0.66 [-1.38 0.05] | 0.06 |
|  | E | 0.40 [0.34 0.47] |  | 1.12 [1.00 1.24] |  |  |  |  |
|  | R | 0.88 [0.76 1.01] |  | 0.61 [0.49 0.72] |  |  |  |  |
| supp − plac | I |  |  |  |  | -20.34 [-45.22 4.53] | -0.83 [-1.84 0.18] | 0.1 |

Table S10. Relative changes of the IL2 / IL10 ratio induced by excursion and by restitution and the differences in these changes between after‑ and before‑training measurement sessions along with Cohen’s d effect size and the probability of hypothesis about equality of their means.

|  |  | before | | |  | after | | |  | after − before | | |
| --- | --- | --- | --- | --- | --- | --- | --- | --- | --- | --- | --- | --- |
| set | change | RC [%] | d | p |  | RC [%] | d | p |  | RC [%] | d | p |
| supp | I-E | -60.46 [-86.32 -34.60] | -1.95 [-2.79 -1.12] | 0.00 |  | 48.23 [22.81 73.64] | 1.59 [0.75 2.42] | 0.00 |  | 108.68 [75.90 141.47] | 2.77 [1.94 3.61] | 0.00 |
|  | I-R | 5.97 [-15.60 27.54] | 0.23 [-0.60 1.07] | 0.53 |  | 31.07 [14.00 48.13] | 1.52 [0.69 2.36] | 0.00 |  | 25.10 [2.79 47.41] | 0.94 [0.1 1.78] | 0.03 |
| plac | I-E | -57.48 [-76.59 -38.37] | -2.15 [-2.87 -1.44] | 0.00 |  | 62.14 [49.27 75.01] | 3.45 [2.74 4.17] | 0.00 |  | 119.62 [96.54 142.70] | 3.71 [2.99 4.42] | 0.00 |
|  | I-R | 22.02 [1.95 42.08] | 0.78 [0.07 1.50] | 0.03 |  | -1.16 [-21.35 19.02] | -0.04 [-0.76 0.67] | 0.90 |  | -23.18 [-47.37 1.01] | -0.69 [-1.4 0.03] | 0.06 |
| supp − plac | I-E |  |  |  |  |  |  |  |  | -10.93 [-47.97 26.1] | -0.3 [-1.32 0.72] | 0.54 |
|  | I-R |  |  |  |  |  |  |  |  | 48.28 [18.05 78.51] | 1.61 [0.6 2.61] | 0.00 |

Table S11. Percentage of Treg in lymphocytes [%] before and after the camp training and its percentage change in initial states caused by this training.

|  |  | before |  | after |  | after − before | | |
| --- | --- | --- | --- | --- | --- | --- | --- | --- |
| set | state | mean |  | mean |  | RC [%] | d | p |
| supp | I | 2.88 [1.33 4.43] |  | 2.68 [1.77 3.60] |  | 3.33 [-37.80 44.47] | 0.07 [-0.77 0.90] | 0.85 |
|  | E | 1.20 [0.64 1.75] |  | 1.59 [0.87 2.31] |  |  |  |  |
|  | R | 4.58 [2.65 6.51] |  | 3.01 [2.15 3.87] |  |  |  |  |
| plac | I | 2.40 [1.68 3.13] |  | 3.08 [2.47 3.68] |  | 28.73 [-10.34 67.81] | 0.53 [-0.19 1.24] | 0.13 |
|  | E | 1.57 [1.10 2.04] |  | 1.78 [1.22 2.34] |  |  |  |  |
|  | R | 4.58 [3.30 5.85] |  | 3.45 [2.34 4.55] |  |  |  |  |
| supp − plac | I |  |  |  |  | -25.4 [26.64 -0.49] | -0.49 [-1.5 0.52] | 0.32 |

Table S12. Relative changes of Treg percentage induced by excursion and by restitution and the differences in these changes between after‑ and before‑training measurement sessions along with Cohen’s d effect size and the probability of hypothesis about equality of their means.

|  |  | before | | |  | after | | |  | after − before | | |
| --- | --- | --- | --- | --- | --- | --- | --- | --- | --- | --- | --- | --- |
| set | change | RC [%] | d | p |  | RC [%] | d | p |  | RC [%] | d | p |
| supp | I-E | -95.33 [-158.56 -32.10] | -1.26 [-2.10 -0.42] | 0.01 |  | -62.03 [-103.41 -20.64] | -1.25 [-2.09 -0.42] | 0.01 |  | 33.31 [-4.87 71.49] | 0.73 [-0.11 1.57] | 0.08 |
|  | I-R | 51.93 [-0.50 104.36] | 0.83 [-0.01 1.66] | 0.05 |  | 12.48 [-17.99 42.96] | 0.34 [-0.49 1.18] | 0.36 |  | -39.45 [-92.80 13.91] | -0.62 [-1.45 0.22] | 0.12 |
| plac | I-E | -45.48 [-101.91 10.96] | -0.58 [-1.29 0.14] | 0.10 |  | -60.19 [-88.08 -32.30] | -1.54 [-2.26 -0.83] | 0.00 |  | -14.71 [-64.68 35.25] | -0.21 [-0.93 0.50] | 0.52 |
|  | I-R | 60.65 [10.91 110.39] | 0.87 [0.16 1.59] | 0.02 |  | 5.58 [-29.42 40.58] | 0.11 [-0.60 0.83] | 0.73 |  | -55.07 [-107.39 -2.75] | -0.75 [-1.47 -0.04] | 0.04 |
| supp − plac | I-E |  |  |  |  |  |  |  |  | 48.02 [-10.13 106.17] | 0.83 [-0.18 1.84] | 0.1 |
|  | I-R |  |  |  |  |  |  |  |  | 15.62 [-52.93 84.18] | 0.23 [-0.78 1.24] | 0.64 |

Table S13. Percentage of NK in lymphocytes [%] before and after the camp training and its percentage change in initial states caused by this training.

|  |  | before |  | after |  | after − before | | |
| --- | --- | --- | --- | --- | --- | --- | --- | --- |
| set | state | mean |  | mean |  | RC [%] | d | p |
| supp | I | 10.28 [4.37 16.18] |  | 7.49 [3.99 10.99] |  | -18.56 [-78.93 41.82] | -0.26 [-1.09 0.58] | 0.49 |
|  | E | 23.36 [18.28 28.43] |  | 13.92 [8.79 19.05] |  |  |  |  |
|  | R | 8.89 [4.46 13.32] |  | 5.54 [1.14 9.95] |  |  |  |  |
| plac | I | 17.71 [12.04 23.39] |  | 9.13 [6.27 11.99] |  | -64.56 [-104.42 -24.71] | -1.16 [-1.87 -0.44] | 0.01 |
|  | E | 24.84 [18.04 31.63] |  | 15.09 [10.31 19.86] |  |  |  |  |
|  | R | 8.85 [3.34 14.35] |  | 5.93 [2.63 9.22] |  |  |  |  |
| supp − plac | I |  |  |  |  | 46.01 [-21.03 113.04] | 0.7 [-0.32 1.73] | 0.16 |

Table S14. Relative changes of NK percentage induced by excursion and by restitution and the differences in these changes between after‑ and before‑training measurement sessions along with Cohen’s d effect size and the probability of hypothesis about equality of their means.

|  |  | before | | |  | after | | |  | after − before | | |
| --- | --- | --- | --- | --- | --- | --- | --- | --- | --- | --- | --- | --- |
| set | change | RC [%] | d | p |  | RC [%] | d | p |  | RC [%] | d | p |
| supp | I-E | 104.63 [45.63 163.64] | 1.48 [0.65 2.32] | 0.00 |  | 65.63 [23.97 107.29] | 1.32 [0.48 2.15] | 0.01 |  | -39.0 [-99.46 21.46] | -0.54 [-1.38 0.30] | 0.17 |
|  | I-R | -3.16 [-54.18 47.86] | -0.05 [-0.89 0.78] | 0.89 |  | -60.49 [-154.86 33.88] | -0.54 [-1.37 0.30] | 0.17 |  | -57.33 [-136.56 21.90] | -0.6 [-1.44 0.23] | 0.13 |
| plac | I-E | 29.57 [-38.35 97.48] | 0.31 [-0.40 1.03] | 0.35 |  | 43.37 [3.30 83.44] | 0.77 [0.06 1.49] | 0.04 |  | 13.8 [-74.57 102.18] | 0.11 [-0.60 0.83] | 0.73 |
|  | I-R | -96.25 [-163.94 -28.56] | -1.02 [-1.73 -0.30] | 0.01 |  | -82.92 [-168.42 2.58] | -0.69 [-1.41 0.02] | 0.06 |  | 13.34 [-34.34 61.01] | 0.2 [-0.52 0.92] | 0.54 |
| supp − plac | I-E |  |  |  |  |  |  |  |  | -52.8 [-152.4 46.8] | -0.54 [-1.55 0.48] | 0.28 |
|  | I-R |  |  |  |  |  |  |  |  | -70.66 [-156.79 15.46] | -0.85 [-1.88 0.19 ] | 0.1 |

Table S15. Percentage of CTL in lymphocytes [%] before and after the camp training and its percentage change in initial states caused by this training.

|  |  | before |  | after |  | after − before | | |
| --- | --- | --- | --- | --- | --- | --- | --- | --- |
| set | state | mean |  | mean |  | RC [%] | d | p |
| supp | I | 8.34 [3.10 13.59] |  | 14.06 [12.38 15.75] |  | 68.35 [25.32 111.38] | 1.33 [0.49 2.16] | 0.01 |
|  | E | 12.31 [6.13 18.48] |  | 12.75 [8.50 17.00] |  |  |  |  |
|  | R | 13.01 [6.84 19.17] |  | 11.55 [8.86 14.23] |  |  |  |  |
| plac | I | 9.72 [7.27 12.17] |  | 17.84 [14.89 20.78] |  | 64.25 [34.41 94.08] | 1.54 [0.83 2.26] | 0.00 |
|  | E | 12.27 [10.70 13.84] |  | 14.34 [12.17 16.50] |  |  |  |  |
|  | R | 13.75 [9.32 18.19] |  | 14.92 [12.84 16.99] |  |  |  |  |
| supp − plac | I |  |  |  |  | 4.1 [52.51 0.09] | 0.09 [-0.94 1.11] | 0.86 |

Table S16. Relative changes of CTL percentage induced by excursion and by restitution and the differences in these changes between after‑ and before‑training measurement sessions along with Cohen’s d effect size and the probability of hypothesis about equality of their means.

|  |  | before | | |  | after | | |  | after − before | | |
| --- | --- | --- | --- | --- | --- | --- | --- | --- | --- | --- | --- | --- |
| set | change | RC [%] | d | p |  | RC [%] | d | p |  | RC [%] | d | p |
| supp | I-E | 43.98 [15.87 72.09] | 1.31 [0.47 2.14] | 0.01 |  | -15.78 [-41.66 10.10] | -0.51 [-1.35 0.33] | 0.19 |  | -59.76 [-92.87 -26.64] | -1.51 [-2.34 -0.67] | 0.00 |
|  | I-R | 51.51 [-6.06 109.07] | 0.75 [-0.09 1.58] | 0.07 |  | -22.16 [-44.40 0.07] | -0.83 [-1.67 0.00] | 0.05 |  | -73.67 [-138.68 -8.66] | -0.95 [-1.78 -0.11] | 0.03 |
| plac | I-E | 27.82 [-7.06 62.70] | 0.57 [-0.14 1.29] | 0.10 |  | -21.58 [-37.55 -5.60] | -0.97 [-1.68 -0.25] | 0.01 |  | -49.40 [-91.36 -7.44] | -0.84 [-1.56 -0.13] | 0.03 |
|  | I-R | 28.77 [-24.89 82.44] | 0.38 [-0.33 1.10] | 0.26 |  | -17.16 [-32.07 -2.24] | -0.82 [-1.54 -0.11] | 0.03 |  | -45.93 [-98.85 6.99] | -0.62 [-1.34 0.09] | 0.08 |
| supp − plac | I-E |  |  |  |  |  |  |  |  | -10.36 [-59.72 39] | -0.21 [-1.22 0.8] | 0.66 |
|  | I-R |  |  |  |  |  |  |  |  | -27.74 [-104.78 49.3] | -0.36 [-1.38 0.65 ] | 0.45 |

Table S17. Percentage of Tδγ [%] before and after the camp training and its percentage change in initial states caused by this training.

|  |  | before |  | after |  | after − before | | |
| --- | --- | --- | --- | --- | --- | --- | --- | --- |
| set | state | mean |  | mean |  | RC [%] | d | p |
| supp | I | 2.38 [1.77 3.00] |  | 3.96 [2.38 5.55] |  | 44.70 [6.70 82.71] | 0.98 [0.15 1.82] | 0.03 |
|  | E | 3.25 [2.02 4.48] |  | 3.46 [2.01 4.91] |  |  |  |  |
|  | R | 2.51 [1.45 3.57] |  | 2.70 [1.59 3.82] |  |  |  |  |
| plac | I | 3.24 [1.91 4.57] |  | 3.88 [2.53 5.22] |  | 22.64 [-4.58 49.87] | 0.60 [-0.12 1.31] | 0.09 |
|  | E | 3.69 [1.79 5.60] |  | 3.42 [2.06 4.79] |  |  |  |  |
|  | R | 2.62 [0.97 4.28] |  | 2.93 [1.66 4.21] |  |  |  |  |
| supp − plac | I |  |  |  |  | 22.06 [65.21 0.52] | 0.52 [-0.5 1.54] | 0.29 |

Table S18. Relative changes of Tδγ percentage induced by excursion and by restitution and the differences in these changes between after‑ and before‑training measurement sessions along with Cohen’s d effect size and the probability of hypothesis about equality of their means.

|  |  | before | | |  | after | | |  | after − before | | |
| --- | --- | --- | --- | --- | --- | --- | --- | --- | --- | --- | --- | --- |
| set | change | RC [%] | d | p |  | RC [%] | d | p |  | RC [%] | d | p |
| supp | I-E | 23.59 [-19.85 67.04] | 0.45 [-0.38 1.29] | 0.24 |  | -19.21 [-47.46 9.04] | -0.57 [-1.40 0.27] | 0.15 |  | -42.80 [-75.29 -10.31] | -1.10 [-1.94 -0.27] | 0.02 |
|  | I-R | -4.71 [-67.19 57.77] | -0.06 [-0.90 0.77] | 0.86 |  | -39.88 [-55.91 -23.85] | -2.08 [-2.92 -1.24] | 0.00 |  | -35.17 [-86.51 16.18] | -0.57 [-1.41 0.26] | 0.15 |
| plac | I-E | 4.21 [-39.87 48.30] | 0.07 [-0.65 0.78] | 0.83 |  | -17.75 [-41.54 6.04] | -0.53 [-1.25 0.18] | 0.13 |  | -21.96 [-68.67 24.74] | -0.34 [-1.05 0.38] | 0.32 |
|  | I-R | -35.98 [-98.98 27.02] | -0.41 [-1.12 0.31] | 0.23 |  | -36.36 [-62.95 -9.77] | -0.98 [-1.69 -0.26] | 0.01 |  | -0.38 [-46.11 45.35] | -0.01 [-0.72 0.71] | 0.99 |
| supp − plac | I-E |  |  |  |  |  |  |  |  | -20.84 [-73.71 32.03] | -0.4 [-1.41 0.61] | 0.41 |
|  | I-R |  |  |  |  |  |  |  |  | -34.79 [-97.88 28.31] | -0.56 [-1.56 0.45 ] | 0.26 |

Table S19. The ratio of Treg/Tδγ percentages before and after the camp training and its percentage change in initial states caused by this training.

|  |  | before |  | after |  | after − before | | |
| --- | --- | --- | --- | --- | --- | --- | --- | --- |
|  | state | mean |  | mean |  | RC [%] | d | p |
| supp | I | 1.19 [0.7 1.69] |  | 0.77 [0.54 1] |  | -41.33 [-87.82 5.16] | -0.74 [-1.58 0.09] | 0.07 |
|  | E | 0.38 [0.21 0.56] |  | 0.55 [0.28 0.81] |  |  |  |  |
|  | R | 2.11 [1.26 2.97] |  | 1.31 [0.84 1.79] |  |  |  |  |
| plac | I | 0.91 [0.56 1.26] |  | 0.97 [0.71 1.22] |  | 10.64 [-37 58.29] | 0.16 [-0.56 0.88] | 0.63 |
|  | E | 0.58 [0.35 0.81] |  | 0.7 [0.34 1.06] |  |  |  |  |
|  | R | 3.02 [1.59 4.45] |  | 2.93 [1.66 4.21] |  |  |  |  |
| supp − plac | I |  |  |  |  | -51.98 [-113.07 9.12] | -0.86 [-1.86 0.15] | 0.09 |

Table S20. Relative changes of Treg/Tδγ ratio induced by excursion and by restitution and the differences in these changes between after‑ and before‑training measurement sessions along with Cohen’s d effect size and the probability of hypothesis about equality of their means.

|  |  | before | | |  | after | | |  | after − before | | |
| --- | --- | --- | --- | --- | --- | --- | --- | --- | --- | --- | --- | --- |
|  |  | RC [%] | d | p |  | RC [%] | d | p |  | RC [%] | d | p |
| supp | I-E | -118.93 [-173.91 -63.95] | -1.81 [-2.64 -0.97] | 0.00 |  | -42.25 [-94.25 9.74] | -0.68 [-1.52 0.16] | 0.10 |  | 76.67 [38.32 115.02] | 1.67 [0.84 2.51] | 0.00 |
|  | I-R | 56.79 [8.27 105.32] | 0.98 [0.14 1.81] | 0.03 |  | 52.14 [17.66 86.62] | 1.26 [0.43 2.1] | 0.01 |  | -4.65 [-65.06 55.76] | -0.06 [-0.9 0.77] | 0.86 |
| plac | I-E | -50.05 [-81.72 -18.38] | -1.13 [-1.85 -0.42] | 0.01 |  | -47.21 [-83.91 -10.51] | -0.92 [-1.64 -0.2] | 0.02 |  | 2.84 [-38.44 44.12] | 0.05 [-0.67 0.76] | 0.88 |
|  | I-R | 96.82 [-2.11 195.75] | 0.7 [-0.02 1.42] | 0.05 |  | 37.06 [-16.22 90.35] | 0.5 [-0.22 1.21] | 0.15 |  | -59.76 [-137.48 17.97] | -0.55 [-1.27 0.17] | 0.12 |
| supp − plac | I-E |  |  |  |  |  |  |  |  | 73.84 [22.08 125.59] | 1.43 [0.43 2.44] | 0.01 |
|  | I-R |  |  |  |  |  |  |  |  | 55.1 [-35.86 146.06] | 0.61 [ -0.4 1.62] | 0.22 |
